# Effectiveness of spirometry as a motivational tool for smoking cessation: a clinical trial, the ESPIMOAT study

**DOI:** 10.1186/1471-2296-14-185

**Published:** 2013-12-05

**Authors:** María Isabel Irizar-Aramburu, Jose Manuel Martínez-Eizaguirre, Petra Pacheco-Bravo, Maria Diaz-Atienza, Iñigo Aguirre-Arratibel, Maria Isabel Peña-Peña, Mercedes Alba-Latorre, Mikel Galparsoro-Goikoetxea

**Affiliations:** 1 Idiazabal Primary Care Medical Centre, Idiazabal, Gipuzkoa, Spain; 2 Villabona Primary Care Medical Centre, Villabona, Gipuzkoa, Spain; 3 Beasain Primary Care Medical Centre, Beasain, Gipuzkoa, Spain; 4 Zumárraga Primary Care Medical Centre, Zumárraga, Gipuzkoa, Spain; 5 Andoain Primary Care Medical Centre, Andoain, Gipuzkoa, Spain; 6 Elgoibar Primary Care Medical Centre, Elgoibar, Gipuzkoa, Spain; 7 Legazpia Primary Care Medical Centre, Legazpia, Gipuzkoa, Spain

**Keywords:** Spirometry, Screening, Smoking, COPD

## Abstract

**Background:**

Smoking is the main preventable cause of morbidity and mortality in our region, it being the main causative agent of chronic obstructive pulmonary disease. There still is no consensus on the use of spirometry as a strategy for smoking cessation, given that there is insufficient scientific evidence from high quality studies to recommend the use of this technique.

**Methods/Design:**

This is to be a randomized, multicentre, open-label clinical trial. A total of 444 smokers over 40 years of age will be recruited by 39 general practitioners from 22 health centers. Primary objective of this study is to assess the effectiveness of spirometry together with information regarding the test for smoking cessation after 1 year in smokers over 40 years of age with a more than 10 pack-year history and no previous diagnosis of chronic obstructive pulmonary disease. Groups of 45 patients who smoke will be randomly selected from the lists of the participating doctors. The names will be sent to the corresponding doctors who will contact candidate patients and assess whether they meet the selection criteria. Patients who meet these criteria will be randomly allocated to an intervention or control group. For patients in both groups, a nurse will conduct an interview and perform a spirometry test to measure forced vital capacity. Then, all patients will be referred for an appointment with their doctor for brief anti-smoking intervention, patients from the intervention group additionally being informed about the result of the spirometry test. After 1 year, smoking status will be assessed and, in those who report that they have quit smoking, abstinence will be confirmed by co-oximetry. Data will be analyzed on an intention-to-treat basis using the chi-squared test for outcomes and binary logistic regression if it is considered to be necessary to adjust for confounding variables.

**Discussion:**

Performing a spirometry test and providing information on pulmonary function may increase awareness of the effect of smoking among smokers who are asymptomatic or have few symptoms and make them decide to quit. Specifically, in patients with chronic obstructive pulmonary disease it might increase levels of motivation to quit smoking in early stages of the disease. If this strategy were to be effective, it could be included in the health promotion activities offered in primary care.

**Trial registration:**

ClinicalTrials.gov Identifier: NCT01821885

## Background

Smoking is the main cause of preventable mortality and morbidity in industrialized countries. It is estimated that in Spain, as many as 56,000 people die every year due to smoking [1]. It is one of the main risk factors for vascular and respiratory diseases and a causative agent for chronic obstructive pulmonary disease (COPD), accelerating the physiological worsening of the lung volume in susceptible smokers [2,3]. COPD is a disease with a high prevalence (10.2% in 40- to 80-year-olds) and a high rate of under-diagnosis (72% of those with COPD not having been diagnosed), according to the EPISCAN study [4]. It is the fifth cause of death in Spain and one of the main causes of morbidity, responsible for a profound worsening in quality of life, especially in advanced stages of the disease [5]. The World Health Organization (WHO) predicts that by 2030, COPD will go from 12^th^ to 5^th^ place in the list of most prevalent diseases in the world, and from 6^th^ to 3^rd^ place in terms of mortality [6]. These data justify the Spanish National Health System launching a strategy against COPD in 2009 [7] focused around smoking cessation interventions.

Smoking is the main cause of COPD, being associated with more than 80% of cases. According to a study by Fletcher et al. [2], 15 to 20% of smokers develop COPD, more recent studies [8,9] arguing that the figure may in fact be as high as 50%. After the results of the Lung Health Study [10] in 1994, a randomized clinical trial that demonstrated that an intensive anti-smoking intervention in patients with mild and moderate COPD managed to halt the decline in FEV1, several consensus statements have recommended spirometry testing in smokers to benefit their general health. In 2000, one of the main sponsors of the screening programs for COPD was the American National Lung Health Education Program (NLHEP) [11] who advocated the use of spirometry for COPD screening in all patients with any respiratory signs and in smokers aged over 45 years old. COPD meets some of the criteria proposed by Frame and Carlson [12] to justify a screening program, that is, it is an important health problem with an long initial asymptomatic stage; there is a screening test with high sensitivity and specificity, namely spirometry, which is considered the gold standard for the diagnosis; and there is a treatment that, if administered in the asymptomatic stage, is effective in reducing overall morbidity and mortality, in this case smoking cessation [2].

Any screening test must have a good cost-effectiveness ratio. The cost-effectiveness of spirometry depends on various factors, such as the number of patients that need to be screened to detect one case of COPD or, better, to result in one person stopping smoking. In the Lung Health Study [10], the most significant results were obtained in middle-aged patients (mean age of 48 years), with mild or moderate COPD (mean post-bronchodilator FEV1 of 78%), most of whom did not consider themselves “ill”: patients from the anti-smoking intervention group had a smaller decrease in FEV1, the authors concluding that spirometry is cost-effective since it permits the early detection of COPD and focusing measures on these patients. Other authors [13] concluded that even modest quit rates attributable to spirometry may be cost-effective, although the authors comment that further studies are required to assess the independent role of spirometry in smoking cessation.

Later, in 2005, the American Agency for Healthcare Research and Quality (AHRQ) published a systematic review [14] concluding that the benefits of spirometry for opportunistic screening to detect COPD in smokers and ex-smokers will remain unclear until it is demonstrated that spirometry contributes to increasing the number of patients who quit smoking. This review included seven clinical trials of which only one [15] assessed the independent contribution of spirometry in smokers and, though they found the quit rate was higher among those who underwent spirometry as well as receiving advice (6.5 vs. 5.5% in those who only received advice), the difference was not significant. The authors of this review also indicated the need for further studies to determine the role of spirometry in smoking cessation. Another systematic review, that of the U.S. Preventive Services Task Force (USPTF) published in 2008 [16], a subsequent systematic review [17] and a 2005 Cochrane review, on the efficacy of assessing biomedical risks (among them pulmonary function) as a tool for quitting smoking [18], all concluded that the evidence of a role of spirometry as a motivational tool to encourage smoking cessation is inconclusive, as most studies had a short follow-up period and significant limitations, such as not assessing the independent role of spirometry and insufficiently large sample sizes.

A 2012 updated review of the Cochrane Collaboration [19] found little scientific evidence of an effect on quitting smoking for most biomedical tests. Out of the 15 studies included, only 2 of them detected a significant effect: a trial based on ultrasound of carotid and femoral arteries and another on spirometry combined with information on test results in terms of “lung age” [20] which found a significant improvement in the smoking cessation success rate compared to the control group that did not receive the spirometry test report (RR 2.12; CI 95% 1.24 to 3.62). Nevertheless, this study still did not assess the independent role of spirometry, as testing was used in both the experimental and control groups.

Based on the available evidence, the executive summary of the Global Initiative for Chronic Obstructive Lung Disease (GOLD) published in 2013 [21], the international reference, the guidelines of the Spanish Society of Family and Community Medicine and the Spanish Society of Pulmonology and Thoracic Surgery (semFYC-SEPAR) [22] and the 2012 Spanish guidelines for COPD (GesEPOC) [23] advocate “opportunistic” case finding by spirometry only in smokers with respiratory symptoms. However, there is no consensus on this strategy in the scientific community, some authors questioning the usefulness of COPD screening in asymptomatic smokers due to the weak correlation between these symptoms in smokers and COPD. A cohort study [24], including 3,955 people in the USA recruited between 1980 and 2008, that attempted to assess the association between respiratory symptoms and airflow obstruction, confirmed the under-diagnosis of airway obstruction in smokers and concluded that respiratory symptoms have low sensitivity, specificity, and positive and negative predictive values for diagnosing COPD, given that these symptoms are common in smokers with and without airway obstruction; on this basis, the authors advocate spirometry testing in all smokers older than 40 years of age with a more than 20 pack-year history of smoking with or without respiratory symptoms.

Therefore, given the available evidence and the lack of high quality clinical trials assessing the independent role of spirometry in smoking cessation, and that discovering they have COPD may help people quit smoking, we believe that the study we propose may be useful to clarify various issues. Specifically, if our hypothesis were to be correct and spirometry testing did help people quit smoking, public health authorities would have a basis on which to promote this strategy, as it would have the potential to change lifestyles and thereby prevent highly prevalent medical conditions such as various types of cancer and cardiovascular disorders, as well as COPD. Additionally, if a significantly higher rate of smokers with an early diagnosis of COPD quit smoking than those with no diagnosis of airway obstruction, the demonstrated beneficial effect in terms of reducing morbidity and mortality due to COPD, would in itself justify the screening. If, on the other hand, no significant effect on smoking cessation were detected, there would be no arguments supporting COPD screening by spirometry, and we should probably stop performing spirometry tests for this purpose, thus avoiding an unnecessary use of resources. The protocol we present here aims to provide evidence to address this issue, by attempting to determine whether conducting spirometry testing to screen for COPD and informing individuals about their results is effective in encouraging smoking cessation in smokers older than 40 years of age with a more than 10 pack-year history.

### Objectives

The primary objective of this study is to assess the effectiveness of spirometry testing together with providing patients with information about their results for smoking cessation after 1 year in people older than 40 years of age with a more than 10 pack-year history who have not been diagnosed with COPD (at the outset of the study). The secondary objectives include comparing the quit rates in patients with a new diagnosis of COPD and those with normal lung function, and comparing the change in daily smoking rate between the intervention and control groups.

## Methods/Design

This is a multicentre, open-label, randomized clinical trial. Study subjects are active smokers older than 40 years of age with a more than 10 pack-year history who have not been diagnosed with COPD, from 39 general practitioner (GP) lists within the province of Gipuzkoa. The following patients will be excluded: any who are older than 80 years of age or institutionalized, as well as any who have a life expectancy of less than 1 year, have undergone spirometry testing within the previous 2 years, have been previously diagnosed with respiratory diseases (asthma, interstitial lung diseases, or COPD) that cause pattern changes in spirometry tests, or have contraindications for spirometry testing (Table 1). This clinical trial has been registered in ClinicalTrials.gov (NCT01821885).

**Table 1:** Definition of the study variables

**Active smoker**	**Diagnosis of chronic obstructive pulmonary disease (COPD)**
Any patient who smokes one or more cigarettes per day will be considered an active smoker.	The criteria for the diagnosis of COPD is a post-bronchodilator FEV1/FVC ratio of < 70% (absolute value).
**Spirometry technique**	**Spirometric patterns**
**Baseline spirometry**: the nurse responsible for the spirometry testing will be experienced in the technique and familiar with the ATS/ERS criteria for acceptability (correct start with back extrapolated volume of <150 ml or 5%, curve with no marks or notches and correct end, with no leaks and lasting at least 6 seconds or with a plateau > 1 second in the volume-time curve).	Spirometry results will be considered within the normal range if FEV1/FVC > 70% (absolute value) and FVC and FEV1 > 80% of the predicted values.
	The pattern is classified as obstructive if FEV1/FVC < 70% (absolute value).
To meet the criteria for repeatability, there must be three acceptable manoeuvres and the FVC and FEV1 values in the two best two must differ by no more than 100 ml.	The pattern is classified as restrictive if FEV1/FVC > 70% (absolute value) and FVC < 80% of the predicted value.
	The pattern is classified as mixed if FEV1/FVC < 70% (absolute value) and FVC < 80% of the predicted value.
**Bronchial challenge:** After performing the baseline test, patients will be given 400 mcg of Salbutamol (in a holding chamber) and 15–20 minutes later a further spirometry test will be conducted applying the same criteria for acceptability and reproducibility as for the baseline test.	The bronchial challenge is regarded as positive when FEV1 or FVC increase by 12% or 200 ml compared to baseline values 15–20 minutes after administering 400 mcg of Salbutamol (in a holding chamber).
**Contraindications for spirometry testing**	**Quitting smoking**
• Poor general health, old age, etc.	A year after the intervention, patients will be considered to have stopped smoking if they report abstinence (defined as no of consumption of tobacco for at least 7 days) and this is confirmed by CO-oximetry (a carbon monoxide concentration in exhaled air of < 10 ppm is considered sufficient).
• Recent pneumothorax (< 6 months)	
• Unstable angina or recent acute myocardial infarction (< 6 months)	
• Recent retinal detachment (< 6 months)	
• Recent abdominal or thoracic surgery (< 6 months)	**Co-oximetry technique**
• Recent eye surgery (< 6 months)	The patient takes a deep breath and holds it for 15 seconds. They then breathe out slowly and completely (as far as they can). It is then necessary to wait for several seconds until the CO-oximeter settles and shows the exact concentration of carbon monoxide (CO) in parts per million (ppm) in the air exhaled by the patient.
• Thoracic aortic aneurysm	
• Hemoptysis of unknown origin	
• Active tuberculosis	
• Tracheostomy	
• Facial paresis	

**Figure 1:**
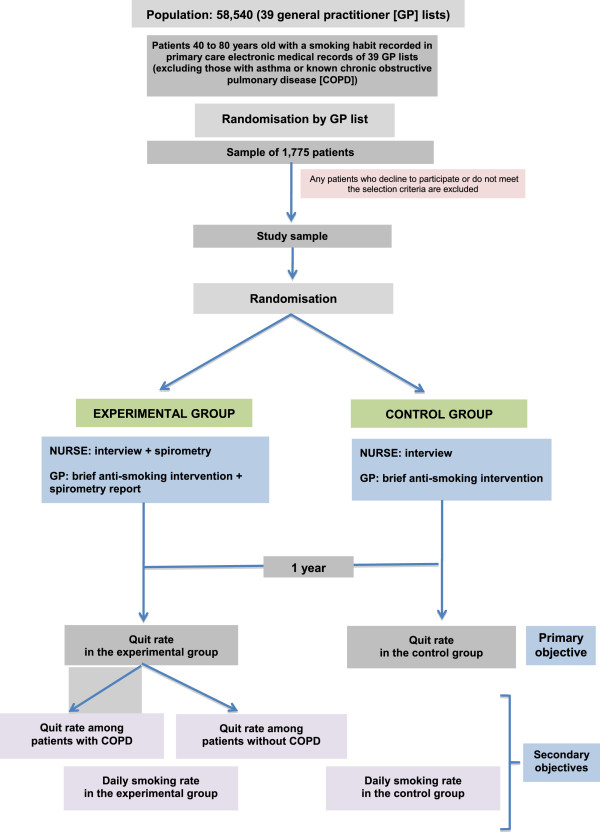
Study flow chart.

A sample of 444 people will be recruited. The sample size calculation was based on assuming a level of significance of 5%, a quit rate after brief anti-smoking intervention only (control group) of 5% [23], an expected quit rate in the experimental group of 14% [20], and a 1:1 ratio of control to experimental subjects. Given these premises, to obtain a power of 80% to detect differences in the test of the null hypothesis Ho: p1 = p2 using a two-tailed chi-squared test for two independent samples, it would be necessary to include 166 experimental units in the reference group and a further 166 in the experimental group, making a total of 332 units for the study. Assuming a loss to follow-up of 25%, it would be necessary to recruit 222 subjects for the reference group and a further 222 for the experimental group, summing to the aforementioned total of 444 subjects for the study. The flow chart for the study is presented in Figure 1.

### Recruitment and randomization

A total of 39 doctors from a research group of an Osakidetza health region within Gipuzkoa are participating in the study. These 39 health professionals work in 22 health centers covering both rural areas (25% of the GP lists) and urban areas, with populations of over 10,000 people (75% of the GP lists).

The total catchment population of these 39 doctors is 58,540 people over 14 years of age. Primary care electronic medical records will be used to identify a sample of these patients who smoke: we will randomly select subjects from the GP lists obtaining a sample of 1775 patients (45 patients per doctor) and each doctor will be informed of the patients in his/her list selected as candidates for the study. After this, the participating doctors will check whether these patients meet the criteria for participating and contact eligible patients by phone to obtain verbal informed consent to their participation in the study and collect preliminary data. Patients will be recruited in a pre-established order from the list provided and the reasons for patient exclusion will be recorded on an individual basis. Doctors will send data on the patients recruited to the Primary Care Research Unit of the Gipuzkoa Health Region where the patients will be randomly assigned to the intervention or control group. The randomization sequence will be generated by computer and kept in the research unit. The list of patients assigned to each group will be sent to the nurses in charge of the first appointment. At this appointment, once written informed consent has been given, the interventions detailed below will be performed for each group.

### Intervention

#### *Experimental group*

The intervention consists of the nurse collecting data, performing a spirometry test and then giving the patient an appointment with their GP within 10 days. At this appointment, the doctor will deliver a brief anti-smoking intervention and provide the patient with a short explanation of the spirometry results. Both the delivery of the advice and the spirometry report will be carried out in a standardised way (in accordance with the guidelines).

#### *Control group*

The nurse will only interview the patient to collect data on the study variables and make them an appointment with their GP within 10 days. The doctor will just perform the brief anti-smoking intervention in the same standardised way as for the intervention group. These patients will not undergo spirometry during the study period of 1 year.

Nurses selected will have the skills and experience necessary for performing spirometry. After finishing this first phase of the study, two doctors with extensive experience in interpreting spirometry will assessed test results and exclude from further analysis any patients whose results do not comply with the criteria for acceptability and repeatability of the American Thoracic Society/European Respiratory Society (ATS/ERS). The nurses involved will not deliver any intervention for smoking cessation to the participants of either group; they will only conduct the interviews and carry out the spirometry, avoiding making any comments on the test results or any other issues related to the patient’s health or smoking habit.

The research doctors will receive training in the study protocol, including how to deliver the brief anti-smoking intervention and report the results of the spirometry test, and will receive a manual with written instructions, summarized in Table 2. In order to avoid variability attributable to the use of different measurement devices, the same models of spirometer (Datospir 120-C) and carbon monoxide detector (Dräger Pac 7000 CO) will be used for all the measurements.

**Table 2:** Intervention by the doctor – instruction manual (summary)

**Brief anti-smoking intervention**	**Information on the spirometry results**
This will consist of a maximum 3 minutes of advice in which the health professional explains clearly to the smoker that the most effective step they could take to improve their health would be to stop smoking and provides them background information in writing. The same material will be provided to all patients and consists of two leaflets on the benefits of stopping smoking (the leaflets being those provided by the Department of Health of the Basque Government and routinely used in primary care consultations for brief anti-smoking interventions).	In the event of spirometry detecting airway obstruction (post-bronchodilator FEV1/FVC < 70%), the patient will be told that they have chronic obstructive pulmonary disease secondary to smoking and that it is very important that they stop their habit.
	In the event of spirometry values being within the normal range, the patient will be told that their respiratory function is not yet impaired and that it would be a good moment to give up smoking.
	In the event of a restrictive spirometry pattern, the patient will be told that they have impaired pulmonary function and they will be provided usual care.
	In all cases, the doctor should address any concerns or queries of the patient concerning the spirometry or any other issues that arise during the consultation.

### Follow-up and assessment of results

All patients will be contacted by telephone 1 and 3 months after the intervention to determine whether they have stopped smoking and, if they have not, how many cigarettes they are smoking per day at the time. One year after the intervention a further phone call will be made by health professionals blinded to the group allocation to assess the patient’s smoking status. For patients that claim to have stopped smoking, the nurse will make an appointment to confirm this using a CO-oximeter that measures the carbon monoxide concentration in exhaled air.

### Study variables

The primary outcome variable will be smoking cessation 1 year after the intervention, as reported by the patient and confirmed by measuring exhaled CO, while the secondary outcome variable will be the number of cigarettes/day for those who continue smoking. Other variables to be assessed include age, sex, body weight and height, BMI, pack-year history, Prochaska’s stage of change, Richmond test score, number of previous attempts to quit smoking, number of periods of abstinence of more than 1 month, use of medication for smoking cessation, respiratory symptoms (coughing, expectoration, degree of dyspnoea), comorbidities (high blood pressure, ischaemic heart disease, cerebrovascular accident, peripheral arterial disease, diabetes), and other habits (alcohol abuse, sedentary lifestyle), as well as spirometry parameters (pre- and post-bronchodilator FVC, FEV1, and FEV1/FVC ratio, predicted FEV1/FVC%). The final outcome measures are to be: CO-oximetry results (in patients who report having stopped smoking), attempts to quit smoking in the previous year, spirometry tests in previous years (excluding that of the study), consultations related to smoking in the previous year (excluding any related to the study), use of medication for smoking cessation in the previous year, use of other methods for smoking cessation in the previous year, and quit rates at 1 and 3 months among those who continue smoking.

Table 1 lists the definitions of the study variables.

### Statistical analysis

Data will be analyzed on an intention-to-treat basis using SPSS 19.0 software. Given that the primary outcome measure is categorical in nature (whether patients do or do not quit smoking), the final comparison will be performed using chi-squared tests and effect size estimates will be expressed as relative risk (RR), absolute risk reduction (ARR), and number needed to treat (NNT) with the corresponding 95% confidence interval (95% CI). Continuous variables will be assessed with the Student’s t-test, if the data meet the assumption of normality, and otherwise with the corresponding non-parametric test.

If this initial analysis demonstrates the need for multivariate analysis, binary logistic regression analysis will be used. This type of model will allow us to compare the primary outcome measure between groups adjusting for any observed differences, and thus minimizing the potential for confounding bias in the final comparison between intervention and control groups. In this case, all the estimations would be expressed as odds ratios with 95% confidence intervals.

### Limitations

Since we will be using an electronic medical record database, any under-recording of smoking habits would mean that some smokers would be excluded from the study. However, according to our internal data, patients’ smoking habits are relatively reliably documented in their primary care medical record. Further, given the type of intervention that will be undertaken, it is not possible to use double blind study design (that would be with blinding of patients and doctors). In order to overcome this, doctors and nurses participating in the study will receive special training, so that the messages they give and their attitudes and behaviors are similar with the two groups (except with regards to delivering the intervention under study), while the second nurse who will assess the smoking status after one year and the person analyzing the results will be blinded to the group allocation of the patients.

### Ethical considerations

The study protocol and informed consent forms were submitted to the Clinical Research Ethics Committee of the Gipuzkoa Health Region and approved, as recorded in the minutes no. 9/2011. The researchers will ensure that the study is conducted in compliance with the principles of the Declaration of Helsinki and the ICH guidelines for good clinical practice and applicable legislation.

All the participants will be informed about the study, its objectives and activities related to their participation, the number of visits, tests to be performed, information on the results, etc. They will be also informed that in the event of any health problem being detected in the course of the study, they will be treated as usual, if necessary being referred to an appropriate unit for diagnosis and treatment, to ensure that they receive the best treatment for their condition. The aforementioned types of information will be provided in writing and individuals will be asked to sign an informed consent form prior inclusion.

## Discussion

The role of primary care in the early diagnosis of COPD is indisputable, as is the need for spirometry testing to diagnose this condition [25]. This technique is currently well established in primary care consultations, the quality of the tests having greatly improved in this level of care [24]. On the other hand, the early diagnosis of COPD by spirometry is still somewhat controversial. Assuming that most of the diagnoses would be mild or moderate COPD, the most important measure to improve morbidity and mortality would be smoking cessation and, as we have noted, very few high quality studies have assessed the independent role of spirometry for achieving this objective[14,16]. Of all the studies identified, only two investigated the independent role of spirometry for smoking cessation and both reached negative conclusions [15,26]. More recently, in 2008, Parkes et al. [20] found that by presenting spirometry results in terms of “lung age” there was a greater decrease in smoking than in a control group who were presented the results in the usual way. Nevertheless, this study does not fully clarify the role of spirometry as testing was performed for both groups. Some previous observational studies [27,28] have found higher quit rates in patients diagnosed with COPD. On the other hand, in order to achieve good results in COPD screening, the increase in quit rates would need go beyond the patients with a new diagnosis of COPD, and extend to smokers in general; this is because if there were higher quit rates in COPD patients without a parallel increase across all the smokers analyzed, the intervention would lose some of its value, since we could assume that the absence of effectiveness were due to the potential reassuring effect for many smokers of discovering that they have “normal” lung function. For this reason, the primary objective of our study is to assess whether performing spirometry and informing patients of the results increases the quit rate in smokers over 40 years of age with a smoking history of more than 10 pack-years, whether they have COPD or not, the secondary objectives being to achieve smoking cessation in those with COPD and decrease the consumption of cigarettes in those who continue to smoke.

Some ethical considerations have been raised about the potential harm of screening labeling patients with the diagnosis of COPD. In a study focusing on this issue [29], 86% of smokers agreed with lung function being measured since this could increase awareness of the negative effects of smoking and increase motivation to give up smoking.

Another potential issue concerns the cost-effectiveness of COPD screening, namely whether it may more effective to carry out spirometry tests on smokers with symptoms as recommended by guidelines [21-23], or on the basis of the number of pack-years smoked. Although, in any case, the objective is to achieve smoking cessation, a change of strategy could be considered depending on the findings of our study, given that other research [30] has demonstrated that the risk of COPD is positively associated with the cumulative smoking index and, on the other hand, there is direct relationship between the intensity of smoking and general morbidity and mortality [31]. As well as the specific objectives of the study, its findings may contribute to determining the percentage of patients who smoke who in fact have COPD, the severity of their condition, and whether the risk of having COPD is greater in patients with symptoms or whether it is more closely related to pack-year history.

## Abbreviations

AHRQ: Agency for healthcare research and quality; ATS/ERS: American Thoracic Society/European Respiratory Society; EPISCAN: The epidemiologic study of COPD in Spain; COPD: Chronic obstructive pulmonary disease; FEV1: Forced expiratory volume in the first second; FVC: Forced vital capacity; GesEPOC: Guía Española de la EPOC (Spanish guidelines on COPD); GOLD: Global initiative for chronic obstructive lung disease; MRC: Medical research council; NLHEP: National lung health education program; SEPAR: Sociedad Española de Neumología y Cirugía Torácica (Spanish Society of Pneumology and Chest Surgery); NNT: Number needed to treat; semFYC: Sociedad Española de Medicina Familiar y Comunitaria (Spanish Society of Family and Community Medicine); USPSTF: U.S. preventive services task force; WHO: World Health Organization.

## Competing interests

The authors declare that they have no competing interests.

## Authors’ contributions

MIIA and JMME proposed the original idea, designed the study protocol, drafted this manuscript, and contributed to the statistical analysis. MIIA, JMME, PPB, MDA, IAA, MPP, MAJ and MGG contributed to developing the conception and design of the study and critically revised the manuscript. All authors read and approved the final version of the manuscript.

## Authors’ information

MIIA: General practitioner, Idiazabal Primary Care Medical Centre, Gipuzkoa, Spain; JMME: General practitioner, Villabona Primary Care Medical Centre, Gipuzkoa, Spain; PPB: General practitioner, Beasain Primary Care Medical Centre, Gipuzkoa, Spain; MDA: Primary care nurse, Zumárraga Primary Care Medical Centre, Gipuzkoa, Spain; IAA: General practitioner, Andoain Primary Care Medical Centre, Gipuzkoa, Spain; MIPP: Primary care nurse, Elgoibar Primary Care Medical Centre, Gipuzkoa, Spain; MAL: General practitioner, Legazpia Primary Care Medical Centre, Gipuzkoa, Spain; and MGG: Primary care nurse, Zumárraga Primary Care Medical Centre, Gipuzkoa, Spain.

## Pre-publication history

The pre-publication history for this paper can be accessed here:

http://www.biomedcentral.com/1471-2296/14/185/prepub
